# Assessing Nurses’ Satisfaction with Continuity of Care and the Case Management Model as an Indicator of Quality of Care in Spain

**DOI:** 10.3390/ijerph18126609

**Published:** 2021-06-19

**Authors:** Gloria Reig-Garcia, Rosa Suñer-Soler, Susana Mantas-Jiménez, Anna Bonmatí-Tomas, Maria Carmen Malagón-Aguilera, Cristina Bosch-Farré, Sandra Gelabert-Viella, Dolors Juvinyà-Canal

**Affiliations:** 1 Department of Nursing, University of Girona, 17003 Girona, Spain; gloria.reig@udg.edu (G.R.-G.); susana.mantas@udg.edu (S.M.-J.); sandra.gelabert@udg.edu (S.G.-V.); 2 Department of Nursing, Health and Health Care Research Group, University of Girona, 17003 Girona, Spain; anna.bonmati@udg.edu (A.B.-T.); carme.malagon@udg.edu (M.C.M.-A.); cristina.bosch@udg.edu (C.B.-F.); dolors.juvinya@udg.edu (D.J.-C.)

**Keywords:** case management, continuity of care, chronic diseases management, nurse management, quality of care

## Abstract

Chronic diseases are treated and cared for in different healthcare settings. Continuity of care and the case management model facilitate the integration of processes and care levels. However, there is little evidence regarding the satisfaction of nurses with this model. The purpose of this study was to examine nurses’ satisfaction with continuity of care and the case management model. A cross-sectional study was conducted. An ad hoc questionnaire was administrated to 437 Spanish nurses from the three health care settings that responded. This included items on socio-demographics, employment relationship, and satisfaction with continuity of care and case management. Descriptive analysis and linear regression models were performed. In total, 96.1% of the nurses expressed a high level of satisfaction with continuity of care and 80.7% with the case management model. Nurses in a primary care setting reported the greatest satisfaction with the case management model (*B* = 0.146, 95% CI = 0.139–0.694, *p* = 0.003). The nurses’ higher perception of patient satisfaction was associated with greater satisfaction with continuity of care (*B* = 0.466, 95% CI = −0.367–0.533, *p* < 0.000). Nurses identified the case management model as an optimal facilitator of continuity of care. While satisfaction with continuity is high, strategies are needed to improve it in primary care centers and aged care homes.

## 1. Introduction

Addressing the global burden of chronic diseases is a major development challenge in the 21st century and is among the priority directives of the World Health Organization (WHO). Increased life expectancy, improvements in public health and healthcare, and the adoption of certain lifestyles indicate that the dominant epidemiological pattern is focused on chronic diseases [1].

These diseases lead to the reduced quality of life of those affected (patients and families), premature mortality, and major economic burdens on families, communities, and society. Chronic diseases are treated and cared for in different healthcare settings, and it has been observed that sometimes patients can feel that they “fall between the cracks” when receiving care in different healthcare settings [2,3]. As a result of these demographic and epidemiological changes, health systems have had to respond to the growing demand for increasingly diversified services and needs [4,5,6].

Hence, there is a need for an environment that adapts to the needs of chronic patients and their co-morbidities, as well as one that brings about a change in the culture among professionals and organizations [7,8]. Continuity of care and case management can provide a solution to this problem [9,10,11].

Continuity of care is the degree of coherence and amalgamation of the experiences that patients perceive over time, so that they are consistent with their health needs and personal circumstances [12]. It can be seen that continuity of care exists when the care provided, maintained over time, and perceived by the patient are in accordance [13], which includes the connection and synchronization of services and the fulfillment of multidisciplinary and person-centered objectives [14,15]. Continuity of care in nursing is based on the transfer of information and the coordination and consistency of care among nurses providing different levels of care, in order to personalize it according to the needs of the patient and the illness involved [16,17]. The continuity of care process is multidisciplinary, but the literature demonstrates that nurses are the key professionals who can improve this process [18,19].

The organizational care model of case management, which originated in Anglosphere countries, stemmed from the necessity to find a balance between the needs of patients and the health system [20]. For this, nurse case managers help the patient/family navigate the system in the most efficient way, minimizing fragmentation and duplication of care, and promoting care integration [21,22]. This model promotes a comprehensive approach to a person in a complex, dependent, and fragile situation, while developing advanced practices [23] and responding to the growing needs of the population. Similarly, the liaison function of nurses ensures the quality and continuity of care at different levels [20]. The case management model reduces hospitalizations, readmissions, medicalization, and functional impairment, and improves the patients’ quality of life [11,24,25].

The Case Management Society of America defines case management as a collaborative process, in which the options and services needed to meet an individual’s health needs are assessed, implemented, coordinated, monitored, and evaluated, bringing together the communication and available resources to promote quality and cost-effective outcomes [9].

Case manager nurses work in the community or hospital environment (nurse case manager, liaison, or advanced practice nurses according to the denomination in the different hospitals or primary care centers). Considering that life expectancy in the region of Catalonia (Spain) is one of the highest in the world, specifically 86.3 years for women and 80.7 for men [26], for this reason, continuity of care and case management programs have been adopted.

Patient safety, quality of care, and nurse-sensitive indicators are common areas of focus in the international research literature relating to patient outcomes [27,28]. In addition, health professionals’ satisfaction with continuity of care and case management models is considered an indicator for their evaluation [29]. However, there is lack of evidence on the nurses’ satisfaction with continuity of care and the case management model in chronic diseases. Therefore, it is important for organizations to identify the satisfaction of nurses, in order to provide quality care for chronic patients who need to be attended to in different settings.

Hence, this study aimed to examine the nurses’ satisfaction and their perceptions regarding continuity of care and the case management model.

## 2. Methods

### 2.1. Design

A cross-sectional study was performed. The study recruited a total of 639 participants (58.1% hospital; 33.6% primary care; 8.3% aged care homes) representing 100% of the nursing professionals working in the participating centers: two hospitals, thirteen primary care centers (PC), and six aged care homes. Only nurses who were working at the time of the study were included.

### 2.2. Instruments

An ad hoc, anonymous, and self-administered questionnaire was developed and adapted to the characteristics of each healthcare setting. It consisted of four sections. First, information regarding sociodemographic and employment variables including age, years since graduation, work experience in this specific healthcare setting, gender, employment relationship, function, training, and research. The second section referred to the nurses’ satisfaction with the continuity of care, and consisted of questions on three aspects: (a) satisfaction with continuity of care; (b) nurses’ perception of patient satisfaction with the continuity of care; and (c) the response time between hospital discharge and first contact with a PC’s nurse as a key element of satisfaction with the continuity of care. Responses to the first two items were measured on a Likert scale (1 = highest satisfaction to 5 = least satisfaction, recoded into a variable of three categories, wherein scores 1 and 2 were high satisfaction, score 3 was medium satisfaction, and scores 4 and 5 were low satisfaction) and the response time was measured in hours.

In the third section, we assessed the nurses’ perception of the need for continuity of care of chronic patients using questions regarding: (a) the need to report and receive information on all nursing activities performed during hospital admission; (b) the need to report and receive information only on activities performed during hospital admission, which require continuity once the patient is discharged from the hospital; and (c) the nurses’ perception that the increase in continuity of care improves the safety of the care provided by nurses. All three questions were measured using a Likert scale (1 = highest satisfaction to 5 = least satisfaction, recoded in a variable of three categories, wherein scores 1 and 2 were high satisfaction, score 3 was medium satisfaction, and scores 4 and 5 were low satisfaction).

The fourth section assessed nurses’ perceptions of case management as part of the current continuity of care model in chronic patients. Two questions were asked regarding: (a) the level of satisfaction with the case management nurse; and (b) satisfaction with the liaison nurse, both measured using a Likert scale (1 = highest satisfaction to 5 = least satisfaction, recoded in a variable of three categories, where scores 1 and 2 were high satisfaction, score 3 was medium satisfaction, and scores 4 and 5 were low satisfaction). Experts from the clinical and methodological fields participated in its preparation.

A validity and reliability test was conducted with 27 nurses. No modifications were required, and all participants showed an understanding of the issues raised and their assessment. We examined internal consistency reliability by calculating Cronbach’s alpha. The Cronbach’s alpha of the subscale of satisfaction was 0.64, and for perception of the need for continuity of care it was 0.81.

### 2.3. Data Collection and Data Analysis

As an anonymous, self-administered questionnaire was used, 639 nurses were invited by the researchers to complete the questionnaire. Subsequently, they were provided with a printed copy that could be completed in approximately 10 min.

Continuous variables were described as the mean and measures of dispersion (standard deviation, median, and interquartile range). Categorical variables were described in terms of absolute frequency and percentage. The bivariate analyses were done by non-parametric Mann–Whitney U test. A multilinear regression analysis was used to study the factors strongly associated with nurse satisfaction.

### 2.4. Ethical Considerations

This study was approved by the management of the 13 primary care centers, two hospitals, and six aged care homes. All participants were informed about the objective of the study. The completed questionnaires did not contain any personal information that could identify the participants. Data were analyzed by a researcher. The ethics’ principles defined in the Declaration of Helsinki were followed.

## 3. Results

### 3.1. Sociodemographic and Employment Characteristics of Participants

The final sample included 437 nurses (68.4%). Of these, 92.4% (n = 404) were women. The average age of the nurses was 40.5 (*SD* 10.7) years. In total, 69.1% (n = 302) had a permanent employment relationship, and 30.9% (n = 135) had a temporary employment relationship. Regarding function, 93.4% (n = 408) were care nurses, 5.1% (n = 22) were management nurses, and 1.5% (n = 7) were liaison nurses or nurse case managers (Table 1).

### 3.2. Nurses’ Satisfaction with Continuity of Care

In total, 96.1% of the nurses expressed high satisfaction with the continuity of care, and 68% perceived that the patients were satisfied with the continuity of care. The youngest nurses, who had recently completed their university studies and were working in the same care setting, were the least satisfied with the continuity of care (Table 2).

Satisfaction with the continuity of care was high for 96.3% of female and 93.9% of male nurses. Nurse perception of patient satisfaction with continuity of care received a higher score among female nurses.

Nurses with temporary contracts were the most satisfied (98.7%) and had the most positive perception of patient satisfaction with the continuity of care (77.6%). Nurses providing care were the most satisfied and had the most positive perception of patient satisfaction with the continuity of care (85.7%).

Depending on the care setting, the nurses who were most satisfied with the continuity of care were those working in hospital care (97.2%), and those who perceived the highest patient satisfaction with the continuity of care were those working in primary care (71.4%) (*p* < 0.05) (Table 2), especially in rural centers (*p* = 0.02).

The average response time between hospital discharge and first contact from the primary care nurse was 28.9 (SD 10.2) hours. Nurses with temporary contracts were those who were considered to have a shorter response time (73.9% before 24 h) and those with permanent contracts were those who had a longer response time (35.9% after 25 h).

Nurses providing care were those with the lowest response time (65.3% less than 24 h) and the response time was highest (66.7% after 25 h) for case management nurses. Nurses providing care to adults reported an earlier contact time (65.1% of adult services nurses said they made contact within 24 h and 27.8% before 36 h), and these differences were statistically significant compared to those working in pediatric care (*p* < 0.05).

### 3.3. Nurses’ Perception of the Need for Continuity of Care

Regarding the nurses’ perception of the need for continuity of care, 95.3% of the nurses agreed on the need to report and receive information on all activities carried out during hospital admission and 97% agreed on the need for only the activities subject to continuity of care. Nurses with permanent contracts considered the need to report and receive information on all activities performed during hospitalization (95.6%) (*p* < 0.05), and above all, on activities subject to continuity of care (96.7%) (*p* < 0.01) (Table 2). Nurses with research training considered it necessary to report only the activities required by continuity of care (90.9%) (*p* < 0.01).

Of the nurses, 92.4% considered that increasing continuity of care promotes the safety of the nursing care provided, with the highest agreement from most experienced professionals (*p* < 0.05).

### 3.4. Nurse Perception of Case Management as Part of the Current Continuity of Care Model

Satisfaction with case management as part of the continuity of care model received a score of 1.76 (*SD* 0.80). Of the nurses, 83.1% were satisfied with the role of the hospital liaison nurse and 78.3% with the role of the primary care case management nurse, relating the satisfaction between the two roles as positive (*p* < 0.00). Younger nurses showed greater satisfaction with liaison nurses and nurse case managers (Table 3). The women rated the case management model more positively (83.4% for women vs. 79% for men; *p* > 0.05). Regarding the care setting, the primary care nurses, especially those from rural centers (*p* < 0.01), were the ones most satisfied with both roles, of the liaison nurse (84%; *p* < 0.05) and the case manager nurse (86.9%; *p* < 0.01) (Table 3). The nurses most satisfied with the role of the liaison nurse were those with a temporary contract (85.8%; *p* < 0.05)*,* a trend that was maintained for the role of the nurse case manager (80.6%; *p* < 0.05) (Table 3).

In the linear regression model, a higher perceived patient satisfaction with the continuity of care was strongly associated with higher nurse satisfaction (*B* = 0.466; *p* < 0.000) (Table 4).

Working in a primary care setting was associated with greater satisfaction with the case management model (*B* = 0.146; *p* = 0.003) (Table 5).

**Figure 1:**
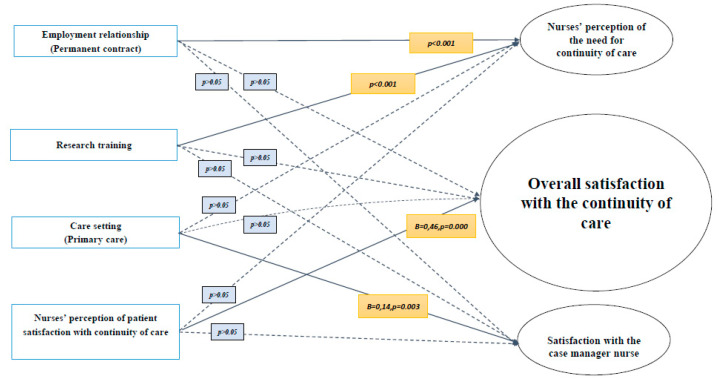
Nurse satisfaction regarding the continuity of care and case management model.

The relationship between satisfaction and the perception of the continuity of care and satisfaction with the case management model, with employment variables and the nurses’ perception of patient satisfaction with the continuity of care is shown in Figure 1.

## 4. Discussion

Health professionals’ satisfaction with continuity of care is considered an indicator for its evaluation [29]. The study showed a high degree of satisfaction with the continuity of care, with no differences based on age and years of professional experience. Previously, the number of years worked had been considered a favorable factor with regard to nursing satisfaction with the continuity of care [30], which depending on the work setting obtained lower scores in aged care homes and PC. Previous studies concluded that there is a widespread perception of a lack of feedback from PC [31], and the existence of continuity of care being due to individual initiative, as it is not recognized and institutionally valued [32].

Patients relate accessibility to the health system and the perception of continuity of care with their level of satisfaction [33,34,35], which increases when they participate in standardized hospital discharge programs [36,37]. Nurse perception of patients’ satisfaction with continuity of care was higher in PC. This could be explained by different reasons. One the one hand, primary care is where chronic patients are attended to and is the most accessible level of care for patients [38]. While, on the other hand, PC is considered the cornerstone of integrating services into a health care system [39].

The response time between hospital discharge and the first contact from the PC nurse has been described as one of the key factors in the continuity of care process. The results of the study showed an average response time less than the 48 h recommended by the Health Department of the studied territory, adding value to the continuity of care process, and with nurses caring for adult patients making contact significantly earlier. Similarly, assuming that pediatric patients have a specific person assigned to them who will be responsible for the liaison function between the levels of care would represent a gap in the safety and quality of the process.

The importance of effective communication between nurses has been pointed out by Jones and Johnstone [40], and it is necessary to increase the motivation and awareness of nurses to communicate at other levels of care [41]. Some nurses do not perceive continuity of care as a direct competence in their profession [42]. Half of the patients readmitted to hospital 30 days after discharge had not been visited by the PC nurse [43]. Regarding nurses’ perception of the need for continuity of care, our results showed the sensitivity of hospital nurses for reporting on the entire hospitalization process and especially aspects with the greatest need for continuity of care. In accordance with this objective, the relationships between a greater perception of the need for continuity of care were significant with the following variables: age, years since graduation, type of contract, and research training. As nurses without research training gave more importance to the need to report activities that require continuity of care, and did not value the need to communicate all aspects related to the process, this leads us to believe that the lack of knowledge of a recurrent theme in research could cause certain aspects of a patient that are important for their comprehensive care to be neglected.

In recent years, patient safety has guided the determination of the quality of all health services. Marek et al. [44] concluded that patients who receive care through a coordination program established between the different levels of care have better results regarding dyspnea, pain, and capacity for undertaking everyday activities. Coinciding with the literature [45,46], the study results point to continuity of care as a fundamental element for ensuring the safety of the nursing care provided.

The case management model is adapted to the context of care, promoting a higher quality of care and facilitating continuity of care in all stages of the process [47]. In this regard, the results of this study identified the case management model as a strategy to facilitate continuity of care. Previous studies have determined that the case management model provides numerous benefits to the patient and the system [23,48]. The application of the model decreases patient readmissions [23,49], improves their therapeutic adherence [50], improves their perception of their quality of life [51], and decreases morbidity in formal caregivers [52]. Moreover, the case management model integrates various providers [53,54], and positively impacts the satisfaction of nurses, who see their role as being more developed [55].

Greater satisfaction with the model as a guarantee of continuity of care has been significantly associated with age and level of care, with the youngest nurses working in primary care being the most satisfied with the case management model; a better understanding of the model provided in university training could explain this result.

Regarding the level of care, the similarity of the role of primary care professionals, who attend to chronic patients comprehensively and longitudinally, and the case management model, could influence the results of greater satisfaction with case management as part of the continuity of care model at this care level. Institutions must make an effort to raise awareness of these new roles among the most experienced groups and professionals working at levels of care other than in primary care.

### 4.1. Implication for Practice

The continuity of care and the application of the case management model facilitate and promote person-centered care and the integration of processes and care levels. It also provides benefits for the patient and the system, as well as developing nursing professionals’ competencies. Knowledge of nurses’ views on the model is important for their development. Primary care is essential for the control and the continuity of chronic patient treatment; however, the results show that primary care nurses are the least satisfied. It would be beneficial to study the variables associated with lower satisfaction and implement strategies to improve it.

A greater homogeneity of the continuity of care model and target populations and the clear description of the role and responsibilities of nurses would clarify model expectations, leading to a refinement of indicators for their evaluation. Therefore, it is necessary to agree on the time devoted to the continuity of care and the ratios of case management nurses.

### 4.2. Study Limitations

First, the main limitation of this study is its cross-sectional design, which only allows for the study of the relationships between variables, without the possibility of establishing causality, even if the size of the study population is appropriate. Moreover, the results reflect the opinions and perceptions at the time of the study, which has limitations when making future projections.

### 4.3. Future Studies

In recent years, multiple and different models of care continuity and case management have emerged. Further research is needed to better understand the relationship between nurses’ satisfaction with different models of continuity of care and case management, and how this affects patient safety and the quality of care, especially for chronic patients.

## 5. Conclusions

Nurses in different care settings show a high degree of satisfaction with the continuity of care model, as well as a high level of perceived need for it. While satisfaction with the continuity of care is high, strategies are needed to improve it among primary care nurses and nurses working in aged care homes. These might include the creation of spaces where nurses from different backgrounds can work together, as networking spaces; increasing the knowledge of nurses about each setting by allowing the rotation of nurses; and shifting certain elements from case management to the continuity of care model.

The case management model is identified as the optimal facilitator in the nursing process of continuity of care, with this perception being greater in primary care. Nurses’ perception of patient satisfaction is strongly related to nurses’ satisfaction with the continuity of care, and the primary care setting is strongly related to satisfaction with the case manager nurse.

## Figures and Tables

**Table 1:** Sociodemographic and employment characteristics of the sample.

	Study Population N: 437
**Age (mean; standard deviation (SD))**	40.5 (10.7)
**Years after completing university studies (University studies) (mean; SD)**	18.1 (10.7)
**Work experience at the same care level (years) (mean; SD)**	14.9 (10.1)
**Gender (n; %)**	
Woman	404 (92.4)
**Employment relationship (Contract) (n; %)**	
Permanent	302 (69.1)
Temporary	135 (30.9)
**Function (n; %)**	
Care	408 (93.4)
Management	22 (5.1)
Liaison nurse or case manager nurse	7 (1.5)
**Training (n; %)**	
Continuous	354 (81.2)
Postgraduate	316 (72.4)
**Research (n; %)**	
Publications in the last 5 years	76 (17.6)
Attended congresses/activities on continuity of care	199 (45.7)

**Table 2:** Nurse satisfaction with the current continuity of care model and related variables.

	**Satisfaction to Report and Receive Information Regarding Hospital Care**				**Satisfaction to Report and Receive Information on the Continuity of Care**				**Satisfaction with the Continuity of Care and the Safety of the Care Provided**			
	**High** **n = 415**	**Medium** **n = 14**	**Low** **n = 8**	***p***	**High** **n = 418**	**Medium** **n = 12**	**Low** **n = 7**	***p***	**High** **n = 404**	**Medium** **n = 24**	**Low** **n = 9**	***p***
**Age (mean; SD)**	40.6 (10.8)	42.8 (10.3)	35.6 (5.9)	0.38	40.5 (10.7)	43.4 (11.3)	36 (5.9)	0.38	40.2 (10.6)	46.1 (11.3)	38.1 (9.5)	0.03
**University studies (mean; SD)**	18.0 (10.8)	20.1 (9.3)	14.7 (9.5)	0.43	18.0 (10.2)	20.8 (11.0)	15.1 (9.9)	0.55	17.7 (10.6)	24.2 (11.1)	17.4 (12.9)	0.03
**Work experience (years) (mean; SD)**	14.9 (10.1)	14.7 (11.9)	12.3 (6.4)	0.89	14.9 (10.1)	18.2 (12.1)	12.7 (6.7)	0.63	14.9 (10)	17.1 (11.5)	11.1 (6.4)	0.50
**Contract (n; %)**				0.02				0,01				0.04
Permanent contract	291 (95.6)	11 (3.9)	1 (0.5)		295 (96.7)	7 (2.8)	1 (0.5)		281 (92.1)	20 (7.1)	2 (0.8)	
Temporary contract	124 (92.5)	3 (2.2)	7 (5.3)		123 (91.8)	5 (3.7)	6 (4.8)		123 (91.7)	4 (3.0)	7 (5.3)	
	**Overall Satisfaction with the Continuity of Care**						**Patients’ Satisfaction with the Continuity of Care**					
	**High** **n = 420**		**Medium** **n = 17**		**Low** **n = 0**	***p***	**High** **n = 297**		**Medium** **n = 127**	**Low** **n = 13**	***p***	
**Age (mean; SD)**	40.6 (10.8)		37.5 (7.7)		-	0.29	39.5 (10.8)		42.1 (10.5)	38.2 (8.9)	0.11	
**University studies (mean; SD)**	18.2 (10.8)		14.9 (9.2)		-	0.33	17.5 (10.7)		19.3 (10.9)	18.0 (10)	0.34	
**Work experience (years) (mean; SD)**	14.9 (10.2)		13.4 (5.4)		-	0.98	14.1 (9.8)		17.0 (10.9)	14.3 (6.8)	0.05	
**Care setting (n; %)**						0.53					0.05	
Primary care	144 (94.1)		9 (5.9)		-		109 (71.4)		42 (27.2)	2 (1.4)		
Hospital care	243 (97.2)		7 (2.8)		-		169 (67.6)		74 (29.6)	7 (2.8)		
Aged care homes	32 (94.1)		2 (5.9)		-		22 (64.7)		8 (23.5)	4 (11.8)		

**Table 3:** Satisfaction with case manager nurses by socio-demographic and employment variables.

	Nurse Satisfaction with the Case Manager Nurse				Nurse Satisfaction with the Liaison Nurse			
	High n = 363	Medium n = 66	Low n = 8	*p*	High n = 342	Medium n = 90	Low n = 5	*p*
**Age (mean; SD)**	40.5 (10.7)	40.2 (10.3)	45.3 (11.9)	0.44	40.5 (10.8)	40.6 (10.4)	41.4 (10.8)	0.97
**University studies (mean; SD)**	17.8 (10.7)	18.8 (13.4)	24.5 (13.3)	0.19	17.7 (10.8)	19.2 (10.3)	19.8 (12.8)	0.45
**Years of experience (mean; SD)**	14.9 (10.1)	15.5 (10.4)	14 (5.5)	0.91	14.8 (10.1)	15.5 (10.2)	12 (8.2)	0.78
**Contract (n; %)**				0.01				0.05
Permanent contract	234 (77.2)	67 (22.1)	2 (0.6)		248 (81.8)	48 (15.8)	7 (2.3)	
Temporary contract	108 (80.6)	23(17.2)	3 (2.2)		115 (85.8)	18 (13.4)	1 (0.7)	
**Care Setting (n; %)**				0.01				0.05
Primary care	133 (86.9)	13 (8.6)	7 (4.6)		129 (84.0)	22 (14.7)	2 (1.3)	
Hospital care	207 (82.8)	42 (16.8)	1 (0.4)		192 (76.8)	55 (22.0)	3 (1.2)	
Age care homes	26 (76.5)	8 (23.5)	-		21 (61.8)	13 (38.2)	-	

**Table 4:** Linear regression model to assess satisfaction with continuity of care nursing (n = 437).

	Dependent Variable: Satisfaction with the Continuity of Care Nursing				
	*B*	*SE*	95% CI	*β*	*p*
Age	−0.006	0.005	−0.015–0.004	−0.084	0.249
Care setting	−0.063	0.053	−0.168–0.043	−0.054	0.243
Years of experience	0.002	0.005	−0.007–0.012	0.033	0.645
Patient satisfaction with the continuity of care	0.450	0.042	−0.367–0.533	0.466	0.000
Satisfaction with the hospital liaison nurse	0.010	0.015	−0.019–0.039	0.031	0.483
Nurse satisfaction with the primary care nurse manager	0.012	0.018	−0.023–0.047	0.029	0.515
*R*2	0.224				
Corrected *R*2	0.213				

*B:* coefficient B; *SE*: standard error; 95% CI: confidence interval of 95%; *β*: standardized beta coefficient. *R*2: *R*-square, the coefficient of determination; corrected *R*2: adjusted *R*-square (adjusted coefficient of determination).

**Table 5:** Linear regression model to assess satisfaction with the case manager nurse (n = 437).

	Dependent Variable: Satisfaction with the Case Manager Nurse				
	*B*	*SE*	95% CI	*β*	*p*
Age	0.015	0.008	−0.033	0.094	0.074
Care setting	0.417	0.141	0.139–0.694	0.146	0.003
Contract	0.149	0.078	−0.305	0.098	0.056
Need to receive information regarding hospital care	0.298	0.125	−0.597	0.115	0.017
*R*2	0.041				
Corrected *R*2	0.032				

*B:* coefficient B; *SE*: standard error; 95% CI: confidence interval of 95%; *β*: standardized beta coefficient. *R*2: *R*-square, the coefficient of determination; corrected *R2*: adjusted *R*-square (adjusted coefficient of determination).

## Data Availability

Not applicable.
